# Calcium-Dependent Chemiluminescence Catalyzed by a Truncated c-MYC Promoter G-Triplex DNA

**DOI:** 10.3390/molecules29184457

**Published:** 2024-09-20

**Authors:** Malay Kumar Das, Elizabeth P. Williams, Mitchell W. Myhre, Wendi M. David, Sean M. Kerwin

**Affiliations:** 1 Materials Science, Engineering, and Commercialization Program, Texas State University, San Marcos, TX 78666, USA; isq12@txstate.edu; 2 Department of Chemistry & Biochemistry, Texas State University, San Marcos, TX 78666, USA; epw14@txstate.edu (E.P.W.); mwm95@txstate.edu (M.W.M.); wdavid@txstate.edu (W.M.D.)

**Keywords:** peroxidase, G-triplex DNA, chemiluminescence

## Abstract

The dynamic landscape of non-canonical DNA G-quadruplex (G4) folding into G-triplex intermediates has led to the study of G-triplex structures and their ability to serve as peroxidase-mimetic DNAzymes. Here we report the formation, stability, and catalytic activity of a 5′-truncated *c-MYC* promoter region G-triplex, c-MYC-G3. Through circular dichroism, we demonstrated that c-MYC-G3 adopts a stable, parallel-stranded G-triplex conformation. The chemiluminescent oxidation of luminol by the peroxidase mimicking DNAzyme activity of c-MYC-G3 was increased in the presence of Ca^2+^ ions. We utilized surface plasmon resonance to characterize both c-MYC-G3 G-triplex formation and its interaction with hemin. The detailed study of c-MYC-G3 and its ability to form a G-triplex structure and its DNAzyme activity identifies issues that can be addressed in future G-triplex DNAzyme designs.

## 1. Introduction

DNA is a multifunctional biopolymer with a range of functions in addition to its role in storing genetic information. Non-canonical DNA structures, such as hairpins, cruciform, H-DNA, i-motifs, Z-DNA, and G-quadruplexes (G4) may play important roles in DNA damage, repair, and genetic instability [1,2]. G4 DNA structures form at telomeric ends as well as in a range of oncogene promoter regions, where they may serve to regulate gene expression [3]. G4 formation in the *c-MYC* promoter nuclease hypersensitive element (NHE) III1 has become a focus for drug targeting by ligands that suppress c-MYC expression through G4 stabilization [4]. In addition to these roles of genomic DNA, DNA oligonucleotides (DNAzymes) can also display a range of catalytic functions [5]. Interestingly, many of these catalytic DNAzymes form non-canonical structures, such as G4 [6,7]. Notably, in the presence of hemin, certain G4 structures can act as peroxidases, catalyzing the oxidation of chromogenic dyes or the chemiluminescent oxidation of luminol [8,9].

**Figure 1:**
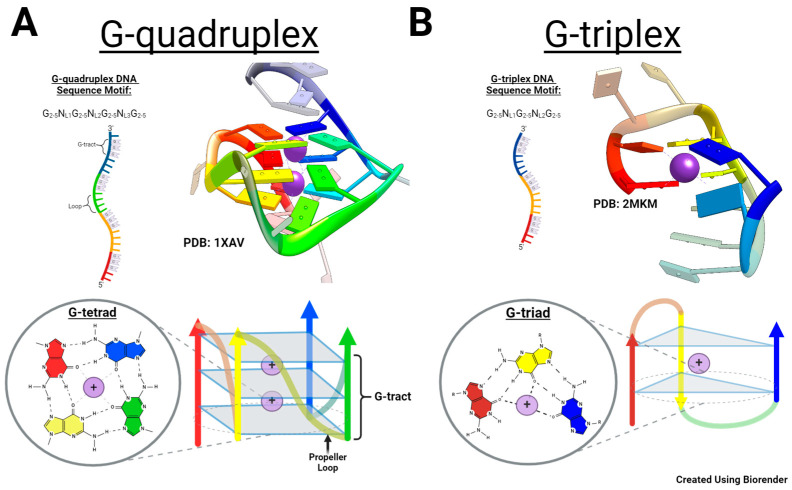
Schematic illustration of DNA structural motifs formed from DNA sequences containing runs of G residues, with the G runs color coded from red to blue from 5′ to 3′ direction. (**A**) Experimentally determined structure of the c-MYC G-quadruplex (PDB: 1XAV) and a cartoon representation of this structure, highlighting the parallel topology of this specific G-quadruplex and the fundamental unit of G quadruplex DNA, the G tetrad. (**B**) Experimentally determined structure of a T1 G-triplex (PDB 2MKM) and cartoon representations of this G-triplex structure, highlighting the antiparallel topology for this specific G-triplex and the fundamental unit of G triplex DNA, the G triad.

G-triplex DNA, which was initially proposed as an intermediate in the process of G4 formation [10], has been recently characterized as a stable structure formed by oligonucleotides with truncated G4 sequences [11]. Functionally, G4 is a complex of four guanine-rich strands with an identified G-tetrad molecular architecture with a planar geometry supported by Hoogsteen hydrogen bonding, whereas G-triplex (G3) structures are three guanine-rich strands associated through triangular G-triads (Figure 1) [11]. Due to the lack of systematic sequence studies, the sequence rules for G-triplex formation are uncertain, and this is complicated by the propensity of G-rich sequences to form non-unimolecular structures. In addition, there is limited data on the direct influence of metal ions or environmental conditions on G-triplex formation. Interestingly, G-triplex DNA has demonstrated peroxidase-like activity, suggesting other functional applications apart from structural functions [12,13,14,15,16,17,18]. 

G-triplex-based biosensors have potential advantages compared to those based on G4 structures. In sensor design, the DNAzyme component is most often masked through hybridization that is disrupted upon aptamer recognition by a ligand [9]. Here, the high thermal stability of G-rich sequences, both in duplex as well as in G4 conformations, can be a liability. For example, the additional G-C base pairs required to hybridize a full G4-forming sequence could make ligand-induced conversion to the G4 more difficult [19,20]. Furthermore, the inherent stability of the resulting G4 limits the conditions under which it may function as a reversible sensor, leading to an extended signal even after the ligand is removed [12,21]. In contrast, G-triplex structures are generally less thermally stable than G4 structures, allowing better coupling between ligand sensing and signal generation. Other potential benefits of G-triplex-based DNAzymes include longer signal duration [18] and shorter sequences required for signal-generation components. However, there have been very limited studies of G-triplex DNAzymes; to date only four G-triplex-forming sequences have been identified that catalyze peroxidase activity [13,15,16,18]. Thus, the true utility and advantages of G-triplex DNAzymes require further detailed studies, including exploration of alternative G-triplex forming sequences.

Here we report on an approach to identify G-triplex structures, starting with the well-studied c-MYC oncogene promoter G4-forming sequence. We introduce SPR as a powerful tool to study G-triplex structures, including interactions with ligands such as hemin, a common co-factor for peroxidase-mimicking DNAzymes. We further show that the G-triplex that we identified catalyzes chemiluminescence oxidation of luminol in a calcium ion-dependent fashion. These studies provide a foundation for the design of improved G-triplex DNAzymes.

## 2. Results and Discussion

**Figure 2:**
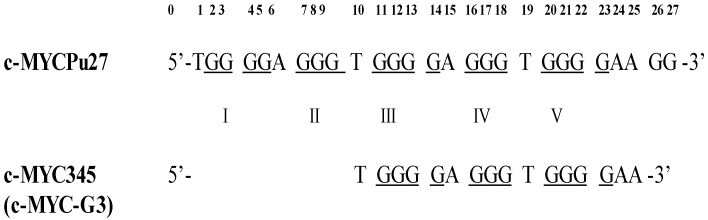
Design of a 5′ truncated G-triplex from c-MYC promoter G-quadruplex. The sequence of the c-MYCPu27 and c-MYC-G3 motifs with G-tracts are underlined.

Since the initial discovery of the G3 via studies on truncations of the telomeric G-quadruplex, reports of stable G-triplexes have been almost exclusively based on truncations of G4 aptamers [20,22]. Considering this, we first set out to establish if a truncated form of the G-quadruplex-forming sequence from the *c-MYC* promoter, c-MYCPu27 [4], can form a stable G-triplex structure. Based on previous kinetic NMR studies on the folding pathway of this sequence [23], we selected a 5′ truncation of c-MYCPu27 containing its three terminal G-tracts, which will henceforth be referred to as c-MYC-G3 (Figure 2). 

### 2.1. Characterization of c-MYC-G3 G-Triplex

**Figure 3:**
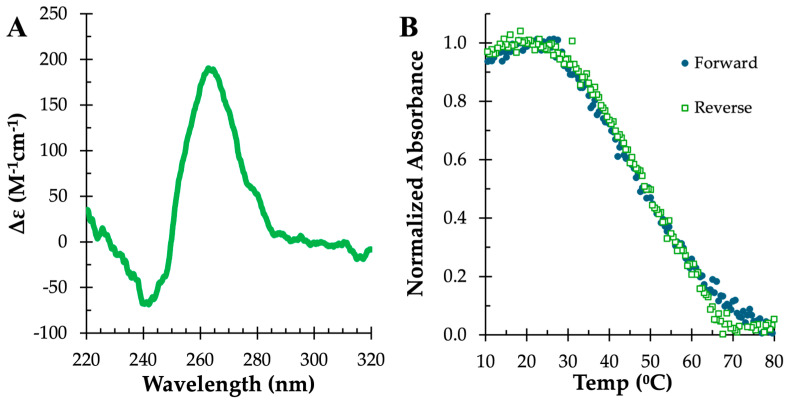
CD and thermal stability of the c-MYC-G3 G-triplex. (**A**) CD spectra of c-MYC-G3 (5 µM) in cacodylate buffer containing 20 mM KCl (pH 7.4) at 25 °C. (**B**) Thermal melts of c-MYC-G3 (5 µM) at 295 nm wavelength in 10 mM Tris buffer (pH 8.0) in both the forward (low to high temperature) and reverse (high to low temperature) directions. The data are presented as average normalized absorbance for forward and reverse melts (n = 2).

To confirm the formation of a stable G-triplex structure by c-MYC-G3, we carried out UV melting studies, CD spectroscopy, and SPR studies. In a K^+^-containing buffer, the CD spectra of c-MYC-G3 displayed a strong, positive CD band at around 265 nm and a negative band at 240 nm that are indicative of a parallel stranded structure (Figure 3A). Similar CD spectra are observed under a variety of conditions for c-MYC-G3 (Figure S1, Supporting Information). Other G-triplex structures that have been established to adopt parallel topology also display similar CD spectra (see Figure S2A, Supporting Information) [24]. In contrast, G-triplex structures that are known to adopt an antiparallel fold display distinct CD spectra [11]. The parallel topology of the c-MYC-G3 G-triplex matches the parallel topology formed by the c-MYC G-quadruplex, from which c-MYC-G3 is derived (See Figure S2B, Supporting Information) [25].

Melting studies were performed to assess the stability of the G-triplex formed by c-MYC-G3. In the absence of added cations, the thermal denaturation of a 5 µM solution of c-MYC-G3 at 295 nm showed a single transition, and the forward and reverse melting curves are nearly superimposable (Figure 3B). In the absence of added cations, the structure formed from c-MYC-G3 is modestly stable, with a Tm of 37–38 °C. 

In order to explore the molecularity of the structure formed by c-MYC-G3, we carried out thermal denaturation studies at c-MYC-G3 concentrations ranging from 5 to 50 µM (Table 1). The melting temperatures at all concentrations were the same, indicating that the structure formed by c-MYC-G3 is unimolecular (Table 1 and Figure S3, Supporting Information). Taken together, the CD and Tm studies indicate that c-MYC-G3 forms a unimolecular G-triplex structure; however, there may be multiple such parallel-stranded structures that are formed by c-MYC-G3 under these conditions. 

It is well established that specific cations are required for the formation and stability of G-quadruplex DNA, with K^+^ and Na^+^ ions being the most studied [26]. Divalent cations may also bind to G-quadruplex structures in a manner similar to that of K^+^ and Na^+^ intercalated between adjacent G-tetrads, or through interactions with the grooves of the DNA, driven by interaction with phosphate groups [26,27]. While the role of cations in the formation and stability of G-triplex DNA is less well studied, studies have suggested that Ca^2+^ ions are preferred to Na^+^, K^+^, and Mg^2+^ ions [28].

To determine the effect of Ca^2+^ on the stability of the c-MYC-G3 G-triplex, the melting temperature experiments were repeated with the addition of CaCl_2_ (3.75 or 7.5 mM). Under these conditions, the Tm increased significantly to 43.4 °C and 51.0 °C, respectively (Table 2). The Tm of c-MYC-G3 in the presence of Ca^2+^ ions is also concentration independent (Table S2, Supporting Information). These findings underscore the binding of Ca^2+^ ions to the G-triplex structure, greatly increasing the stability of c-MYC-G3 in the presence of calcium ions.

Surface plasmon resonance (SPR) is a technique in which biomolecular interactions may be measured in real time and in a label-free manner. By first immobilizing one binding partner to the surface of a gold chip, then injecting the other partner over the chip surface at different concentrations, the resulting changes in refractive index, proportional to mass, near the chip’s surface can be plotted as a function of time to derive equilibrium and kinetic binding constants. A typical strategy for DNA immobilization exploits the high-affinity streptavidin–biotin interaction to capture biotinylated oligonucleotides on the surface of a gold chip that has been pre-functionalized (via amide coupling to a carboxymethyl dextran matrix) with streptavidin [29]. SPR has been utilized to study G-quadruplexes in a variety of ways, including folding kinetics, protein and helicase interactions, and small molecule binding [30,31,32]. However, reports of SPR investigations of G-triplex DNA have been limited to a single study utilizing DNA-peptide conjugates designed to impose a G3 structure [33]. Given that the spatial constraints imposed by the streptavidin DNA capture method limit the ability of immobilized DNA molecules to interact with one another, SPR offers a means to study putative G-triplex sequences that precludes multimer formation. 

**Figure 4:**
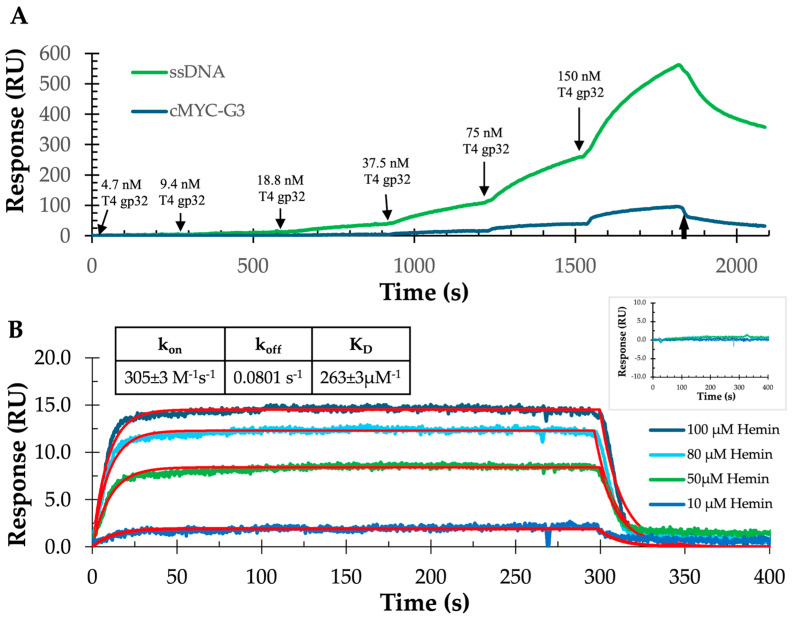
SPR investigation of the formation of the c-MYC-G3 G-triplex and its affinity for hemin. (**A**) A kinetic titration of 4.7–150 nM T4 gp32 SSB protein was performed over flow cells hybridized with equivalent amounts of single-stranded DNA or c-MYC-G3. Arrows pointing downward indicate injection start times, and the upward pointing arrow indicates the end point of the final injection (**B**) Hemin injections over c-MYC-G3 and ssDNA (inset) hybridized flow cells. Data for the c-MYC-G3-hemin sensorgrams were fit to a 1:1 binding model (red lines). The kinetic dissociation constant for the binding of hemin to the c-MYC-G3 G-triplex was determined as the ratio of *k_on_*/*k_off_*. Inset: Minimal binding was observed between hemin and single-stranded DNA. All experiments were conducted in a running buffer composed of 10 mM HEPES and 3 mM EDTA (pH 7.4), with 150 mM LiCl, 10 mM CaCl_2_, and 0.05% *v*/*v* surfactant P20.

Single-stranded DNA binding (SSB) proteins have previously been used to probe G-quadruplex formation in SPR [34]. Here, SSB proteins were used in a similar manner to verify G-triplex formation on the SPR chip surface. Following immobilization of equivalent amounts of c-MYC-G3 and ssDNA in separate flow cells, a kinetic titration of T4 gp32 SSB protein was performed to probe for structure formation in the presence of various ions. To minimize non-specific interactions and favor DNA hybridization necessary for immobilization [35,36], ion concentrations were increased to up to 150 mM monovalent cation and up to 10 mM CaCl_2_. Lithium chloride was included with CaCl_2_ due to its decreased propensity to promote G4 structure formation [26]. Multiple injections of increasing concentrations of T4 gp32 over the flow cell containing single-stranded DNA demonstrated robust binding of the SSB (Figure 4A, green trace). In contrast, there was little change in the flow cell containing immobilized c-MYC-G3 compared to that of single-stranded DNA (Figure 4A, blue trace). This is indicative of structure formation by c-MYC-G3 under these conditions.

Having established the formation of the G-triplex form of c-MYC-G3 by SPR, we next investigated the ability of hemin to bind to this G-triplex, as this is a prerequisite for the peroxidase-mimicking activity for this G-triplex. Hemin injections were performed on a chip functionalized with c-MYC-G3 on one flow cell and single-stranded DNA on a separate flow cell. Unlike single-stranded DNA (ssDNA), which showed no response to the hemin injections, c-MYC-G3 exhibited a discernible dose-dependent response that was fit to a 1:1 binding model to obtain an equilibrium dissociation (*K_D_*) constant of 263 ± 3 µM (Figure 4B). No binding of hemin to single-stranded DNA was observed (Figure 4B, inset). These results both confirm the folding of c-MYC-G3 into a G-triplex structure and establish the affinity of this G-triplex for hemin. Previous studies using bio-layer interferometry analysis demonstrated no interaction between various anti-parallel, mixed parallel/anti-parallel, or intermolecular G-quadruplexes and hemin [37]. The ability of the c-MYC-G3 to bind hemin is commensurate with the parallel topology of the G-triplex as determined by CD (Figure S2A). However, the affinity of c-MYC-G3 for hemin determined here is notably lower than that reported for the parallel c-MYC G-quadruplex DNA (*K_D_* = 1.5 µM) [37]. The fewer stacking interactions between hemin and G-triplets as compared to G-tetrads, and a lower stability of G-triplex compared to G-quadruplex, may contribute to the lower binding affinity for hemin for this G-triplex compared to c-MYC G4.

### 2.2. Characterization of c-MYC-G3 G-Triplex as a Peroxidase-Mimicking DNAzyme through Chemiluminescence

**Figure 5:**
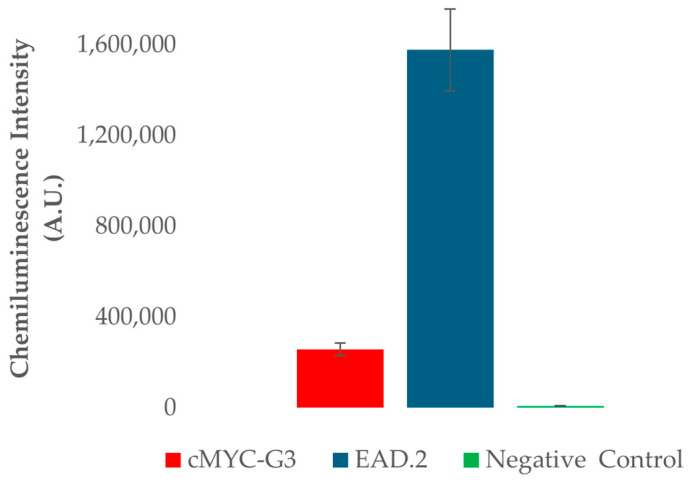
The chemiluminescence catalytic activity of c-MYC-G3 G-triplex. c-MYC-G3 or the G4-forming EAD.2 (1 µM) were incubated with hemin (0.25 µM) and luminol (25 µM) in Tris buffer (10 mM Tris, 20 mM KCl, 200 mM NaCl), pH 8.0, and the intensity of chemiluminescence was measured 1 min after the addition of H_2_O_2_ (13 mM). For the negative control, all reagents except DNA were used.

We subsequently performed a luminol-based chemiluminescence assay to evaluate the peroxidase-mimicking function of the c-MYC-G3 G-triplex in comparison to that of a well-established G-quadruplex chemiluminescence catalyst, EAD.2 [38]. In order to compare c-MYC-G3 to this G4 DNAzyme, we employed conditions previously reported for G4 DNAzyme chemiluminescence, with hemin concentrations several orders of magnitude lower than our calculated *K_D_* for hemin binding c-MYC-G3. In the absence of Ca^2+^ ions, c-MYC-G3 in the presence of hemin catalyzes the chemiluminescent oxidation of luminol (Figure 5). However, under these conditions, the chemiluminescence activity of c-MYC-G3 is approximately 6-fold lower than that of the G-quadruplex EAD.2 (Figure 5). Thus, in the absence of added cations, conditions in which c-MYC-G3 forms a weakly stable G-triplex structure (Tm = 37–38 °C, vide infra), it is a relatively weak catalysis for chemiluminescence. Negligible chemiluminescence is observed in the absence of G-triplex or G-quadruplex DNA (Figure 5) or in the presence of DNA that can not adopt a G-triplex or G-quadruplex structure (Figure S4, see Supporting Information).

**Figure 6:**
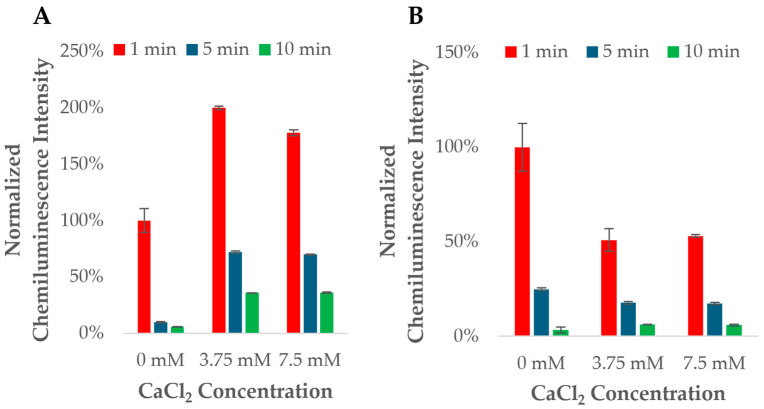
Ca^2+^ ions enhance the chemiluminescence of c-MYC-G3 based DNAzyme. DNAzyme assay consisted of truncated c-MYC-G3 sequence (**A**), and EAD.2 (**B**) with varying concentrations of CaCl_2_ (0 mM, 3.75 mM, 7.5 mM) in 25 mM Tris buffer, pH 8.0, 0.05 µM hemin, 5 µM luminol, 0.0005% Triton X-100, and 1.3 mM H_2_O_2_.

As Ca^2+^ can stabilize c-MYC-G3, we examined the effect of Ca^2+^ on the chemiluminescence. In the presence of 3.75 mM Ca^2+^, the chemiluminescent signal due to the c-MYC-G3 G-triplex increased approximately two-fold compared to the activity in the absence of added Ca^2+^ (Figure 6A). In contrast, the EAD.2 G4 chemiluminescent activity was reduced to about half its original when the concentration of Ca^2+^ was increased (Figure 6B). The duration of the chemiluminescent signal for c-MYC-G3 is greater in the presence of Ca^2+^ than in its absence. In the presence of 3.75 mM CaCl_2_, 10 min after initiation, the signal is approximately 20% of that at 1 min, whereas, in the absence of added Ca^2+^, the signal at 10 min is nearly zero (Figure 6A). This prolonged chemiluminescence signal for c-MYC-G3 in the presence of Ca^2+^ is also slightly longer that of the G-quadruplex EAD.2; the signal intensity for EAD.2 at 10 min is only 11% that at 1 min (Figure 6B). Further increase of the Ca^2+^ concentration to 7.5 mM had only minor effects on both the c-MYC-G3 and EAD.2 chemiluminescence activity (Figure 6). 

**Scheme 1:**
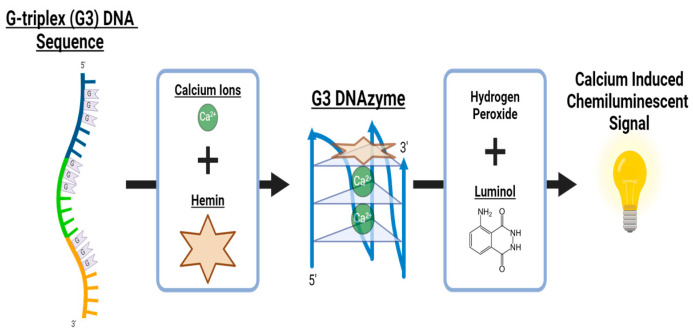
Schematic representation of the effect of Ca^2+^ ions on c-MYC-G3 chemiluminescence. In the presence of Ca^2+^ ions, c-MYC-G3 (left, colored blue to orange 5′ to 3′ direction) forms a stable G-triplex that binds hemin. The resulting complex catalyzes oxidation of luminol with H_2_O_2_ to provide a chemiluminescence signal.

Based on this data, we propose that increased stabilization of the c-MYC-G3 in the presence of Ca^2+^ leads to more efficient catalysis (Scheme 1). The Ca^2+^-bound form of c-MYC-G3 is more stable to thermal denaturation than that formed in its absence, and we propose that it is also more stable to degradation under peroxidase catalysis, leading to enhanced chemiluminescence duration. The exact mode of Ca^2+^ binding by the c-MYC-G3 G-triplex is not known; in Scheme 1, the Ca^2+^ is shown binding to G-triads, similar to the proposed binding of K^+^ by an antiparallel G-triplex [11]. However, other modes of interaction are also possible, similar to the case of divalent metal ions binding to G4 DNA [26]. In contrast to the enhancement of c-MYC-G3 chemiluminescence in the presence of Ca^2+^, the diminished chemiluminescence with increased Ca^2+^ observed for EAD.2 may be due to an unfavorable conformation or destabilization in the presence of Ca^2+^ that leads to lowered catalysis. Thus, despite the diminished affinity of c-MYC-G3 for hemin compared to that reported for parallel G-quadruplex, the c-MYC-G3 G-triplex is nearly equivalent to the G-quadruplex EAD.2 as a chemiluminescence DNAzyme in the presence of millimolar concentrations of Ca^2+^ under these conditions.

**Figure 7:**
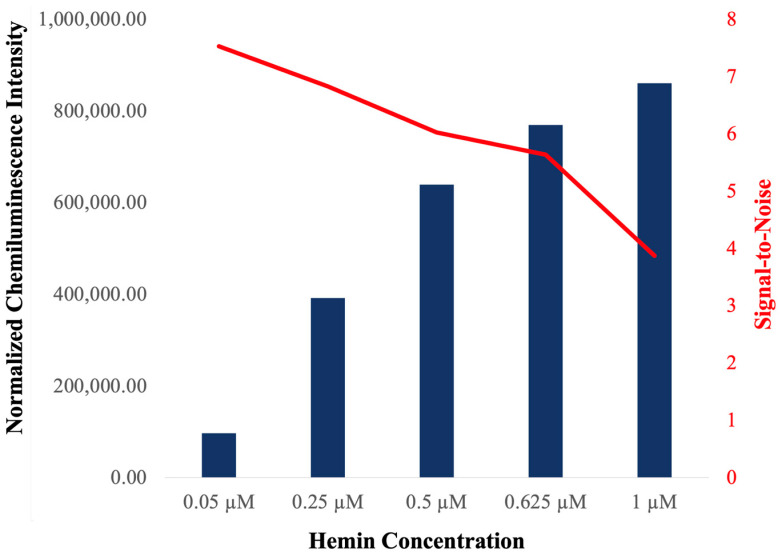
Effect of hemin concentration on c-MYC-G3 catalyzed chemiluminescence in the presence of 3.75 mM CaCl_2_. The chemiluminescence signal is plotted as a function of hemin concentration, plotted as bars, and the signal-to-noise (chemiluminescence in the presence of c-MYC-G3/chemiluminescence in the absence of DNA) is plotted as a red line. Conditions: Tris buffer (25 mM), 3.65 mM CaCl_2_, pH 8.0, c-Myc-G3 (1 µM), luminol (0.5 µM), Triton X-100 (0.0005%), and H_2_O_2_ (1.3 mM).

Having established that Ca^2+^ is required for robust chemiluminescence by c-MYC-G3, and in light of the SPR hemin binding study results above (Figure 4B), we next looked at the effect of increasing the hemin concentration at a fixed 3.75 mM concentration of CaCl_2_ (Figure 7). As expected, there is an increase in chemiluminescence with increased hemin concentration; however, the background signal also increases with hemin concentration, resulting in a steady decrease in the signal-to-noise (S/N) (Figure 7). We selected a hemin concentration of 0.5 µM as a good compromise between signal intensity and S/N. At this hemin concentration, the chemiluminescence intensity is over 6-fold higher that that obtained at 0.05 µM.

**Figure 8:**
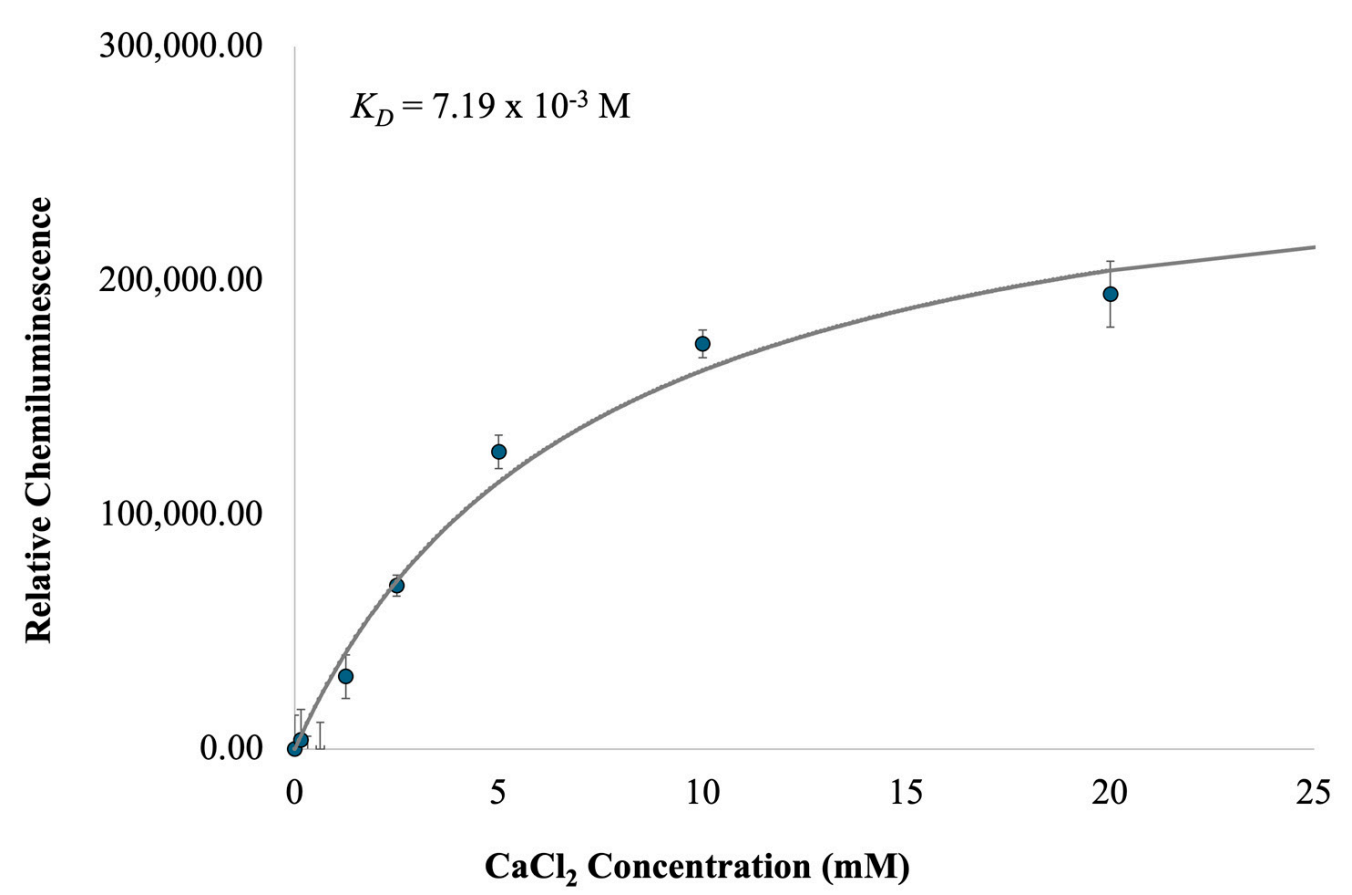
Impact of Ca^2+^ concentration on c-MYC-G3 catalyzed chemiluminescence. The chemiluminescence signal corrected for that determined at 0 mM CaCl_2_ is plotted as a function of CaCl_2_ concentration. Data were fit to a simple single-site binding equation (see Materials and Methods). Conditions: Tris buffer (25 mM), pH 8.0, hemin (0.5 µM), luminol (5 µM), Triton X-100 (0.0005%), and H_2_O_2_ (1.3 mM).

Given the critical role of Ca^2+^ in the chemiluminescence efficiency of the c-MY-G3 G-triplex, we analyzed the effect of Ca^2+^ in more detail. The chemiluminescence signal generated by c-MYC-G3 in the presence of 0.5 µM hemin was determined at Ca^2+^ concentrations between 0 and 20 mM (Figure 8). The chemiluminescence demonstrates saturation with increasing CaCl_2_, and the data were fit to a binding isotherm with a calculated *K_D_* of 7.19 mM (Figure 8). This is significantly higher than the 34 µM Ca^2+^ dissociation constant reported for a human telomeric G-triplex [28]; however, a different G-triplex designed to bind thioflavin T is not stabilized by Ca^2+^ [24]. Thus, there appears to be a variation in the Ca^2+^ binding ability for different G-triplex structures. It is also possible that the relatively high Ca^2+^ concentration that leads to saturation of the chemiluminescence signal observed in Figure 8 is a result of a combination of Ca^2+^ binding affinity and the effect of Ca^2+^ on the catalytic activity of c-MYC-G3, for example, through interaction between the bound hemin and Ca^2+^. In fact, we observe variations in the response of c-MYC-G3 chemiluminescence to Ca^2+^ ion concentration at different hemin concentrations, indicative of the interaction between hemin and Ca^2+^ in peroxidase activity (see Figure S8, Supporting Information).

## 3. Materials and Methods

### 3.1. Experimental Reagents and Materials 

The HPLC purified DNA sequences (Table 3) were obtained from Integrated DNA Technologies (Coralville, IA, USA). Black polystyrene, Costar 96 well assay plates were obtained from Corning Inc. (Corning, NY, USA). All reagents were obtained commercially and used without further purification. 

### 3.2. Apparatus and Software 

Chemiluminescence was measured using a BioTek Synergy neo2 multi-mode plate reader. CD spectra were acquired on a Jasco J-710. UV melting curves were obtained on a Jasco V-730 UV-Visible Spectrophotometer, fitted with a PAC-743R Automatic 6-Position Peltier Cell Changer. SPR experiments were carried out on a Biosensing Instruments BI-2500 benchtop SPR system using streptavidin-functionalized (SA) sensor chips. Binding sensorgrams were analyzed using Biosensing Instrument BI-2500 SPR Data Analysis software version 3.8.5 accessed on 9 August 2024.

### 3.3. Circular Dichroism

c-MYC-G3 samples (5 µM) in 20 mM cacodylate with 20 mM KCl (pH 7.4) were annealed at 90 °C for 5 min followed by cooling to 10 °C over 160 min. before measurement. A 1 cm long quartz cuvette was utilized to acquire measurements. The CD spectra were obtained across 8 scans, using the following parameters: 0.5 nm data interval, 1 nm band width, 100 nm/min scanning speed, and 4 seconds response time. The scans were conducted in 220–320 nm wavelength range. The buffer contribution was eliminated from each sample spectra. Excel was used for final data analysis.

### 3.4. UV Melting Temperature Analysis

Solutions of c-MYC-G3 (5 or 20 µM) in 10 mM Tris buffer (pH = 8 at 25°C), 0.0005% Triton X-100, and CaCl_2_ (0 mM, 3.75 mM, 7.5 mM) in 1.0 mm quartz cuvettes were heated to 90 °C in the spectrophotometer. Absorbance measurements were taken at 260 nm and 295 nm wavelengths as the temperature was decreased by 0.5 °C/min to 10 °C. The samples were heated once more to 90 °C using identical parameters. Melting points (Tm) were determined by applying the baseline median technique [39] using Jasco Spectra Manager software version 2.07.00 accessed on 8 August 2024.

### 3.5. SPR Chip Setup

**Scheme 2:**
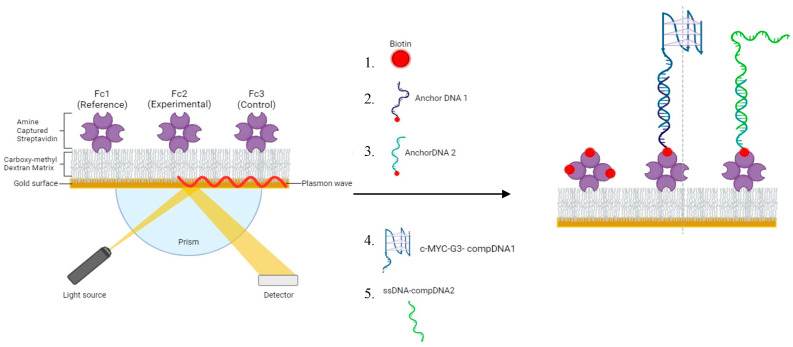
Procedure of c-MYC-G3 biosensor preparation. Isolated injections of biotin, anchor DNA 1, and anchor DNA 2 on each of the three flow cells Fc1, Fc2, and Fc3, respectively, was followed by injections of the c-MYC-G3-compDNA1 and ssDNA-compDNA2 over all flow cells, leading to selective hybridization of c-MYC-G3 on flow cell 2 (Fc2) and ssDNA on flow cell 3 (Fc3). All ligand injections were prepared in immobilization buffer composed of HBS-EP (pH = 6.5) supplemented with 300 mM LiCl and 20 mM CaCl_2_. Immobilization and hybridization steps were conducted at a flow rate of 10 µL/min, while regeneration steps were conducted at a flow rate of 150 µL/min. Immobilization estimates were made by measuring the increase in baseline response from immediately before anchor DNA injection to after final regeneration injections and equilibration.

HBS-EP running buffer (pH 7.4, 10 mM HEPES, 3 mM EDTA, and 0.05% surfactant P20, supplemented with 30–320 mM of specified salt) was used for all experiments. All buffer and reagents were prepared in sterile milliQ water, filtered through a 0.22 µm filter, thoroughly degassed prior to addition of P20 and used in the instrument. Chip setup was conducted using an adapted protocol [40] and is fully outlined in Scheme 2. Briefly, a biosensor chip composed of a reference (subtraction) channel in flow cell one, an experimental (G3) channel two, and a control (ssDNA) flow cell in channel three was constructed for binding studies. The reference flow cell was first blocked off via 5–10-min injections of 5 uM biotin in flow cell one in standard plumbing configuration (Figure S5). Anchor DNA immobilizations were conducted via 2–10-min injections of 1 µM biotinylated DNA, aiming for equal immobilization levels (~400–600 RU) in each flow cell. Several regeneration injections (1M LiCl) were conducted between immobilization and plumbing reconfiguration to remove any loosely bound DNA from the chip’s surface, to minimize the cross-contamination between flow cells and ensure immobilization measurements were accurate. Exclusive capture of anchorDNA1 in flow cell two was enabled through reconfiguring the instrument’s plumbing configuration to the “I-1” setup (Figure S6) and exclusive capture of anchorDNA2 in flow cell three was enabled via the “reverse” setup (Figure S7). Following immobilization and regeneration in flow cell three, plumbing was returned to the standard configuration for subsequent hybridization and analyte injections. 

### 3.6. SPR Binding Experiments

Immediately before each binding experiment, complementary DNAs (in immobilization buffer) were injected over all channels via 5-min injections over all three flow cells, with hybridization of c-MYC-G3 and ssDNA occurring exclusively in flow cells two and three, respectively. All analyte injections occurred at a flow rate of 50 µL/min, over all three flow cells. All binding sensorgrams are single referenced to flow cell one. 

T4 gp32 binding experiments were conducted in standard HBS-EP running buffer (pH 7.4, 10 mM HEPES, 3 mM EDTA, and 0.05% surfactant P20) supplemented with 150 mM LiCl, 10 mM CaCl_2_. Dilutions of analyte (150 µM, 75 µM, 37.5 µM, 18.8 µM, 9.4 µM, 4.7 µM) were prepared in running buffer and injected using a single-cycle kinetic method. This method consists of sequential injections of analyte at increasing concentrations, without a dissociation phase or regeneration steps to clear the chip between injections [41,42]. Samples were prepared via serial dilution of T4 gp32 in running buffer. Each injection consisted of a 5-min association time, and the final injection (150 mM T4 gp32) included a 5-min dissociation phase. Regeneration was conducted via 1–2-min injections of 3X detergent solution (0.3% (*w*/*w*) CHAPS, 0.3% (*v*/*v*) surfactant P20, 0.3% (*v*/*v*) Triton X-100), or 500 mM LiCl/20 mM LiOH solution. The surface was considered fully regenerated when all compDNA and protein were successfully removed from the cell surface, as measured by a decrease in baseline response to below pre-hybridization levels.

c-MYC-G3–hemin experiments were conducted in HBS-EP running buffer (pH 7.4, 10 mM HEPES, 3 mM EDTA, and 0.05% surfactant P20) supplemented with 150 mM LiCl, 10 mM CaCl_2_, and 1% DMSO) at a flow rate of 50 µL/min, using the standard multi-cycle kinetic injection method. Contact and dissociation times were 300 s and 180 s, respectively. Gradient concentration (10 µM, 50 µM, 80 µM, 100 µM) samples of DMSO-solubilized hemin diluted in running buffer were injected in all flow cells at escalating concentrations. Regeneration was conducted using 1 M LiCl when hybridized DNA could be preserved. Following injections of higher concentrations of hemin, stronger regenerations (3X detergent and 500 mM LiCl/20 mM LiOH solutions) were required to fully remove hemin from the chip’s surface, at the expense of hybridized DNA. The binding rate constants of c-MYC-G3–hemin interactions were determined by a non-linear analysis of the association and dissociation curves with the SPR kinetic evaluation program BI 2500. The data were fitted using the 1:1 model. The association and dissociation rate constants, *k_on_* and *k_off_*, were determined. Finally, the kinetic dissociation constants were determined from the binding rate constants: *K_D_* = *k_on_*/*k_off_*.

### 3.7. Chemiluminescence Assays 

The chemiluminescent oxidation of luminol by DNAzymes was evaluated in 96-well plate assays. Oligonucleotides c-MYC-G3 or EAD.2 in 50 mM Tris buffer, pH 8.0, and 0.1% Triton X-100 with variable added KCl/NaCl (40 mM and 400 mM, respectively) or CaCl_2_ (0–7.5 mM) were heated to 88 °C for 5 min in a heat block and then permitted to anneal to room temperature. Subsequently, in black, flat, 96-well plates, the DNA was incubated with 0.25 µM hemin for another hour. After completion of the incubation, 25 µM luminol (25 µM stock solution in 10% DMSO) was added along with 13 mM H_2_O_2_ (13 mM stock solution). The final concentrations in the well included 1 µM DNA, 0.05 µM hemin, 0.0005% Triton X-100, 5 µM luminol (2% DMSO), and either KCl/NaCl (20 mM and 200 mM, respectively) or varying concentrations of CaCl_2_ (0 mM, 3.75 mM, or 7.5 mM), 1.3 mM H_2_O_2_, and 25 mM Tris, pH 8.0. The plate was then read at intervals of one, five, and ten minutes using a BioTek Synergy neo2 multi-mode plate reader. Data for triplicate wells were averaged.

Fitting of the data on the effect of CaCl_2_ on chemiluminescence was carried out using Equation (1) on GraphPad Prism (https://www.graphpad.com/) to afford fitted values for K_d_ = 7.19 × 10^−3^ M (95% confidence interval = 4.12–13.42 × 10^−3^ M) and B_max_ = 278,009 (95% confidence interval = 219,715–373,106) with R^2^ = 0.9756.

Signal = [B_max_ × Conc.]/K_d_ + Conc.] (1)

## 4. Conclusions

In the current work, we investigated the formation and characteristics of c-MYC-G3, a 5′-truncated c-MYC promoter G-quadruplex sequence. c-MYC-G3 adopts a stable G-triplex conformation with a parallel topology, as demonstrated by circular dichroism spectroscopy. The presence of calcium ions considerably increases the thermal stability of c-MYC-G3. We show that SPR is a powerful tool in the study of putative G-triplexes and their interactions with ligands. Using SPR, we confirmed the intramolecular formation of the c-MYC-G3 G-triplex and its ability to bind hemin. Furthermore, our work uncovered the peroxidase-mimicking DNAzyme activity of c-MYC-G3 in the presence of calcium ions through chemiluminescence by oxidation of luminol. This work highlights the influence of metal ions in the catalytic ability of G-triplex structures and the benefits of optimizing conditions to afford robust chemiluminescence by G-triplex DNA.

The study of truncated c-MYC sequence c-MYC-G3 and its ability to form a G-triplex structure is significant, given the intense interest in non-canonical DNA structures and their role in the transcriptional control of c-MYC. The field of G-triplex DNAzymes is young, with few specific examples of G-triplex DNAzymes applied to sensing applications. Thus, there are many issues that have yet to be addressed, such as metal ion effects, efficiency, and signal duration. The identification of the peroxidase activity leading to chemiluminescence catalyzed by c-MYC-G3 provides insights on the DNAzyme activity of this G-triplex. This represents a significant expansion of the number of G-triplex motifs that are shown to catalyze peroxidase activity, and this will provide more flexibility in sensor design. Furthermore, we show some potential advantages of c-MYC-G3 over more widely explored G4-based peroxidase mimics, including a longer duration of the chemiluminescence signal and a unique response to Ca^2+^ ions. We have also identified issues that can be addressed in future G-triplex DNAzyme design. One issue is the relatively weak binding of hemin by c-MYC-G3, compared to that of G-quadruplex DNAzymes, which can be addressed to some extent through increases in hemin concentration. Ultimately, identification of G-triplexes with increased affinity for hemin may lead to more efficient DNAzymes. The effect of Ca^2+^ on the stability and efficiency of c-MYC-G3 chemiluminescence indicates that metal ion effects should also be considered in future efforts to design and characterize G-triplex-based DNAzymes. 

## Figures and Tables

**Table 1:** Effect of c-MYC-G3 concentration on melting temperature (Tm).

c-MYC-G3 Concentration	Tm ^a^
5 µM	38.4 ± 1.1 °C
20 µM	37.7 ± 0.4 °C
30 µM	37.4 ± 0.5 °C
50 µM	37.2 ± 1.0 °C

^a^ Tm for c-MYC-G3 in 10 mM Tris buffer (pH 8.0) as determined at 295 nm. Values are averages and standard deviations of 3 determinations.

**Table 2:** Effect of CaCl_2_ concentration on the Tm of c-MYC-G3 ^a^.

Conditions	0 mM CaCl_2_	3.75 mM CaCl_2_	7.5 mM CaCl_2_
Tm (°C)	38.4 ± 1.1	43.4 ± 1.2	51.0 ± 0.7

^a^. Tm for c-MYC-G3 in 10 mM Tris buffer (pH 8.0) as determined at 295 nm. Values are averages and standard deviations of 3 determinations.

**Table 3:** Oligomers used in the study.

DNA Name	Sequence (5′ to 3′)
EAD.2	CTG GGA GGG AGG GAG GGA
c-MYC-G3	TGG GGA GGG TGG GGA A
anchorDNA1	BioTEG TTTTTTTT GAG CAG CAA TAC ACG A
anchorDNA2	BioTEG TTTTTTTT ACA GGA TCT GCA TCT C
c-MYC-G3- compDNA1	TGG GGA GGG TGG GGA A TTTTTTTT TCG TGT ATT GCT C
ssDNA-compDNA2	TGT GTA GTG CGG GTT TTTTTTTT GAG ATG CAG ATC CTG T

## Data Availability

The data presented in this study are available on request from the corresponding author.

## Supplementary Materials

The following Supporting Information can be downloaded at: https://www.mdpi.com/article/10.3390/molecules29184457/s1, Table S1: Sequences of all DNA oligonucleotides used; Figure S1: CD spectrum of C-MYC-G3 under differing ionic conditions; Figure S2: Comparison of CD spectra for parallel-stranded G-triplex and c-MYC G4 structures; Table S2: Concentration effects on the Tm of c-MYC-G3 in the presence of CaCl_2_; Figure S3: Concentration independence of c-MYC-G3 melting temperature; Figure S4: ssDNA control chemiluminescence; Figure S5: Default flow cell configuration of the BI-2500; Figure S6: I-1 flow cell configuration of the BI-2500; Figure S7: Reverse flow cell configuration of the BI-2500; Figure S8: Hemin concentration-dependent effect of CaCl_2_ on c-MYC-G3 chemiluminescence.
