# Supplementary material for: Calcium-Dependent Chemiluminescence Catalyzed by a Truncated c-MYC Promoter G-Triplex DNA
Source: Molecules. 2024 Sep 20;29(18):4457. doi: 10.3390/molecules29184457 (PMC11434422; doi:10.3390/molecules29184457)
Supplement: Supplementary file 1 [file molecules-29-04457-s001.zip › molecules-3180037-SI.pdf]

REVISED

Calcium-Dependent Chemiluminescence Catalyzed by a Truncated c-MYC Promoter G-Triplex DNA

Malay Kumar Das[1], Elizabeth P. Williams[2], Mitchell W. Myhre[2], Wendi M. David[2], and Sean M. Kerwin[1,2,*]

[1] Materials Science, Engineering, and Commercialization Program, Texas State University, San Marcos, TX 78666, USA; isq12@txstate.edu
[2] Department of Chemistry & Biochemistry, Texas State University, San Marcos, TX 78666, USA; epw14@txstate.edu (E.P.W.); mwm95@txstate.edu (M.W.M.); wdavid@txstate.edu (W.M.D.)
* Correspondence: smk89@txstate.edu; Tel.: +1-5122452056

Supporting Information

Table S1. Sequences of all DNA oligonucleotides ………………………………………… S2
Figure S1. CD spectrum of c-MYC-G3 under different ionic conditions……………………... S3
Figure S2. Comparison of CD spectra of parallel topology G-triplex G31, the c-MYC-G3 G-triplex, and full-length c-MYC-Pu27 G-quadruplex…………………………………………… S3
Figure S3. Effect of DNA concentration on Tm of c-MYC-G3……………………………… S4
Table S2. Effect of DNA concentration on Tm of c-MYC-G3 in the presence of Ca^{2+} ……… S4
Figure S4. G-triplex is required for chemiluminescence…………………………………..... S5
Figure S5. Flow system of the BI-2500 SPR in default configuration..…………………..…... S6
Figure S6. Flow system of the BI-2500 in I-1 plumbing configuration…………………...…... S7
Figure S7. Flow system of the BI-2500 in reverse configuration…………………………..…… S8
Figure S8. Hemin concentration-dependent effect of Ca^{2+} on chemiluminescence………..… S9

Table S1. Sequence of all DNA Oligonucleotides

Number	Name	Sequence
1	EAD.2	5′-CTG GGA GGG AGG GAG GGA-3′
2	c-MYC-G3	5′-TGG GGA GGG TGG GGA A-3′
3	anchorDNA1	5′-BioTEG TTTTTTTT GAG CAG CAA TAC ACG A-3′
4	anchorDNA2	5′-BioTEG TTTTTTTT ACA GGA TCT GCA TCT C-3′
5	c-MYC-G3-compDNA1	5′-TGG GGA GGG TGG GGA A TTTTTTTT TCG TGT ATT GCT C-3′
6	ssDNA-compDNA2	5′-TGT GTA GTG CGG GTT TTTTTTTT GAG ATG CAG ATC CTG T-3′
7	CompDNA1-G31	5'-TCG TGT ATT GCT GCT CTT TTT TTT TGG GTA GGG CGG G-3′
8	c-MYCPu27-345T-compDNA1	5′-TGG GGA GGG TGG GGA ATT TTT TTT TCG TGT ATT GCT C-3′
9	cMYC22mA	5'-TGA GGG TGG GTA GGG TGG GTA A-3′
10	Comp1s	5′-TCG TGT ATT GCT GCT CTT TTT TTT-3′

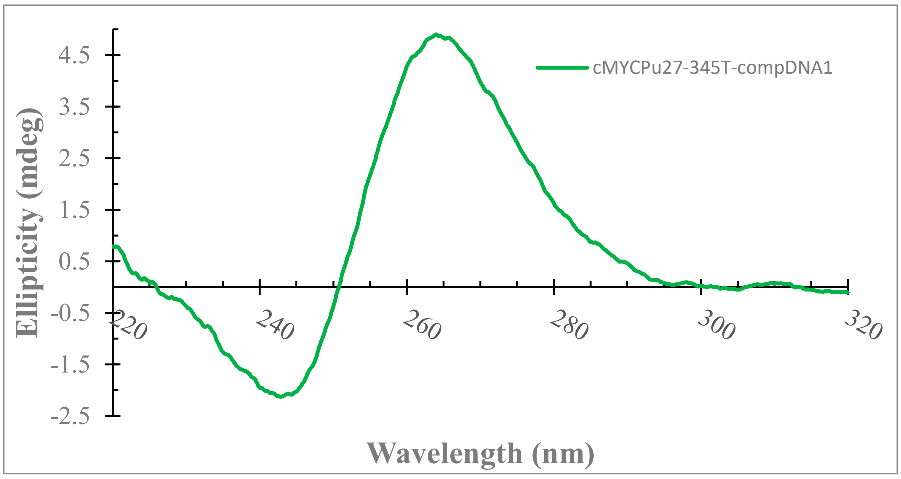

Figure S1. CD spectra of MYC-G3-compDNA1 (oligonucleotide 5) at 5 µM DNA concentration in in buffer containing 200 mM KCl, 15 mM $MgCl_2$ (pH 6.5) at 25 °C with the signal for buffer and comp1s (oligonucleotide 10) subtracted.

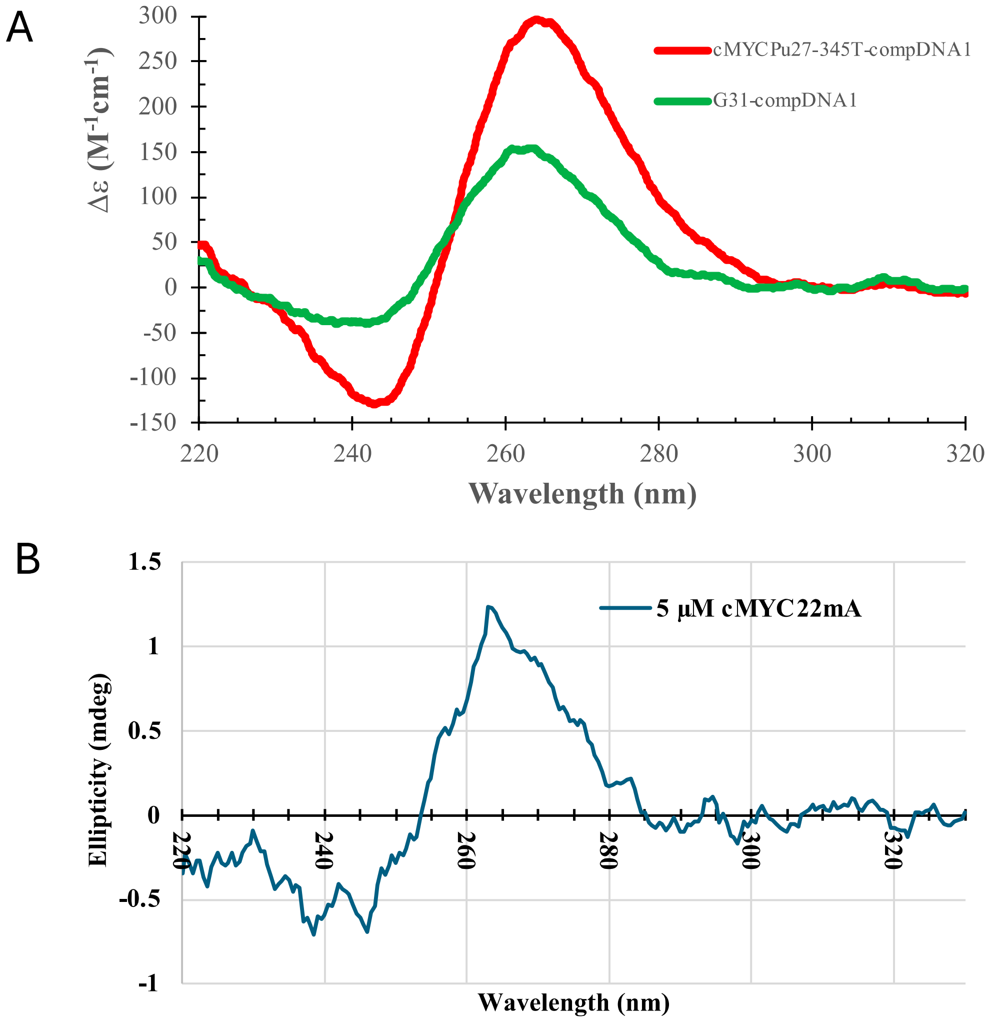

Figure S2. Comparison of CD spectra of G-triplexes and G-quadruplex. (A) green line: CD spectrum of parallel-stranded G31-comp DNA1 (oligonucleotide 7); red line: CD spectrum of c-MYC-G3-compDNA1 (oligonucleotide 5). Both spectra were determined at 5 µM DNA concentration in SPR immobilization buffer (10 mM HEPES, 200 mM KCl, 15 mM $MgCl_2$, 3 mM EDTA, 0.005% surfactant P20) with the signal for buffer and comp1s (oligonucleotide 10) subtracted. (B) CD spectrum of parallel-stranded G-quadruplex cMYC22mA in SPR immobilization buffer.

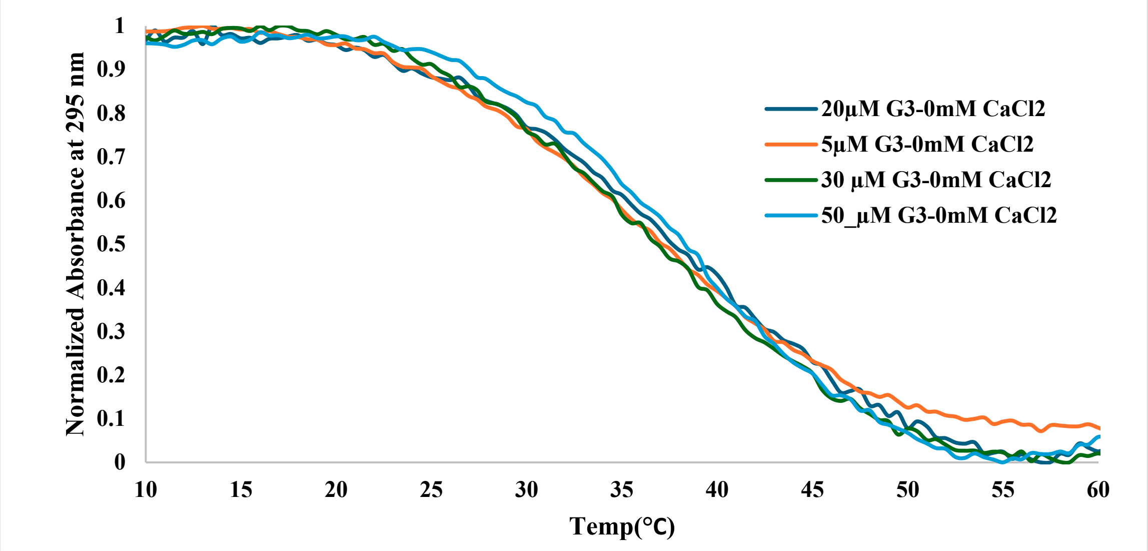

Figure S3. Effect of DNA concentration on Tm of c-MYC-G3. Melting curves for various concentrations of c-MYC-G3 (5 to 50 µM). Conditions: Tris buffer (10 mM, pH 8.0). Tm values are reported in **Figure 3C.**

Table S2. Effect of DNA concentration on the T_m of c-MYC-G3 in the presence of Ca^{2+} ions.[a]

c-MYC-G3 Concentration	Tm
5 µM	40.9 °C
20 µM	40.5 °C
50 µM	41.7 °C

a. Conditions: Tris buffer (10 mM, pH 8.0) with 2 mM $CaCl_2$. T_m values determined at 295 nm.

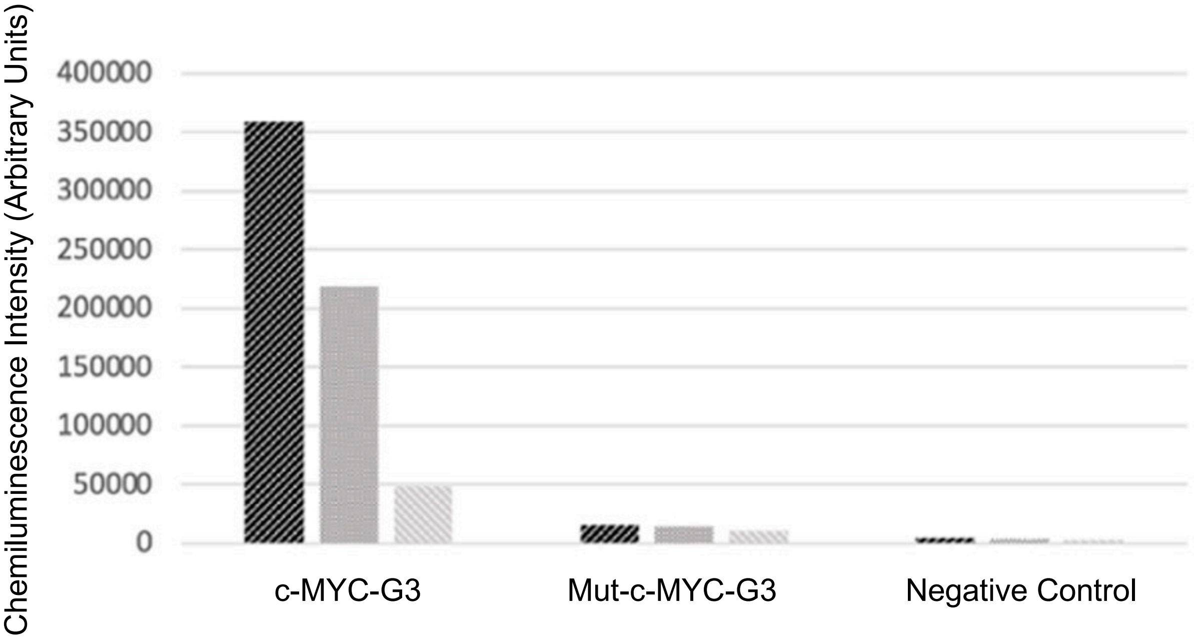

Figure S4. G-triplex is required for chemiluminescence. The chemiluminescence catalytic activity of c-MYC-G3 G-triplex (1 μM), or a mutated sequence incapable of forming G-triplex structure, Mut-c-MYC-G3 (*5'- TGT GTA GTG CGG-3'*) (1 μM) were incubated with hemin (0.25 μM) and luminol (25 μM) in Tris buffer (10 mM Tris, 20 mM KCl, 200 mM NaCl) pH 8.0 and the intensity of chemiluminescence measured 1 min after the addition of H_2O_2 (13 mM). For the negative control, all reagents except DNA were used.

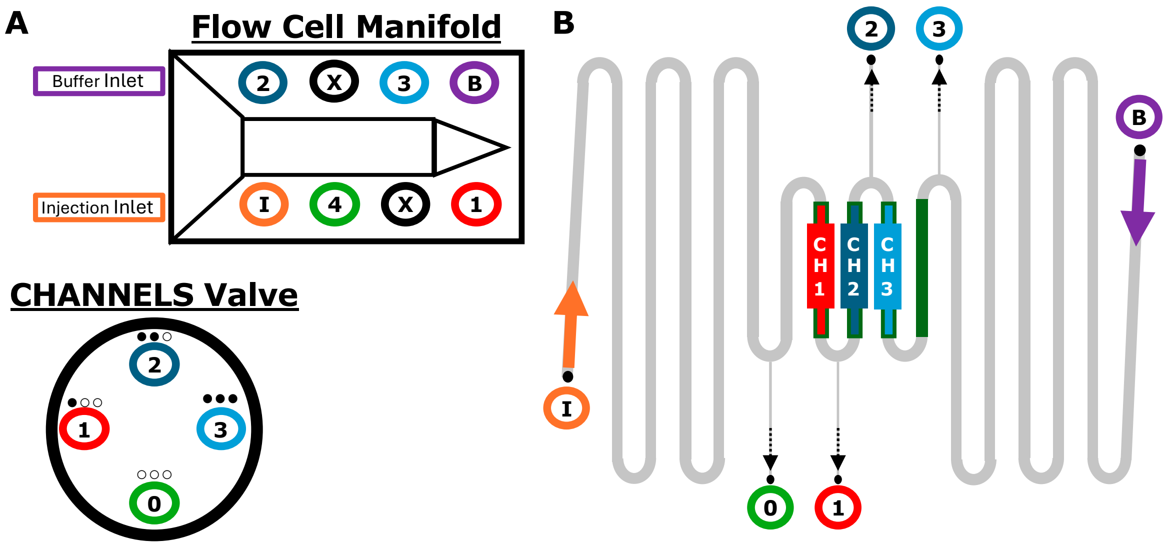

Figure S5. Flow system of the BI-2500 in default configuration. (A) Plumbing schematic of the BI-2500 SPR system showing connections between the CHANNELS switch valve and the flow cell manifold in setup one. (B) Schematic of the flow cell showing the SPR channels CH1, CH2, and CH3. Injection and buffer flow in opposite directions from their respective inlets, meet at the junction of the selected outlet (based on CHANNELS valve position), and exit as waste.

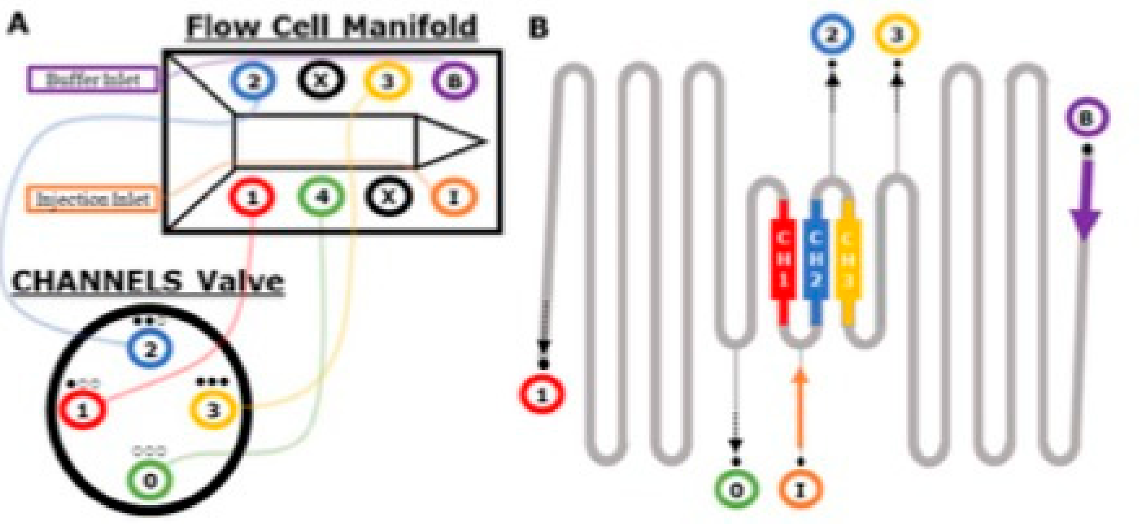

Figure S6. Flow system of the BI-2500 in I-1 plumbing configuration**.** (A) Plumbing schematic of the BI-2500 SPR system after switching lines to I-1 configuration, showing connections between the CHANNELS switch valve and the flow cell manifold (B) Schematic of the flow cell showing the SPR channels CH1, CH2, and CH3. Injection and buffer flow in opposite directions from their respective inlets. If CHANNELS valve is in position 2 or 3, the flows meet at the junction of the selected outlet and exit as waste, while fluid over CH1 remains stagnant. If the CHANNELS valve is set to position 0 or 1, the flows meet at the junction between CH1 and CH2, and flow to selected outlet together.

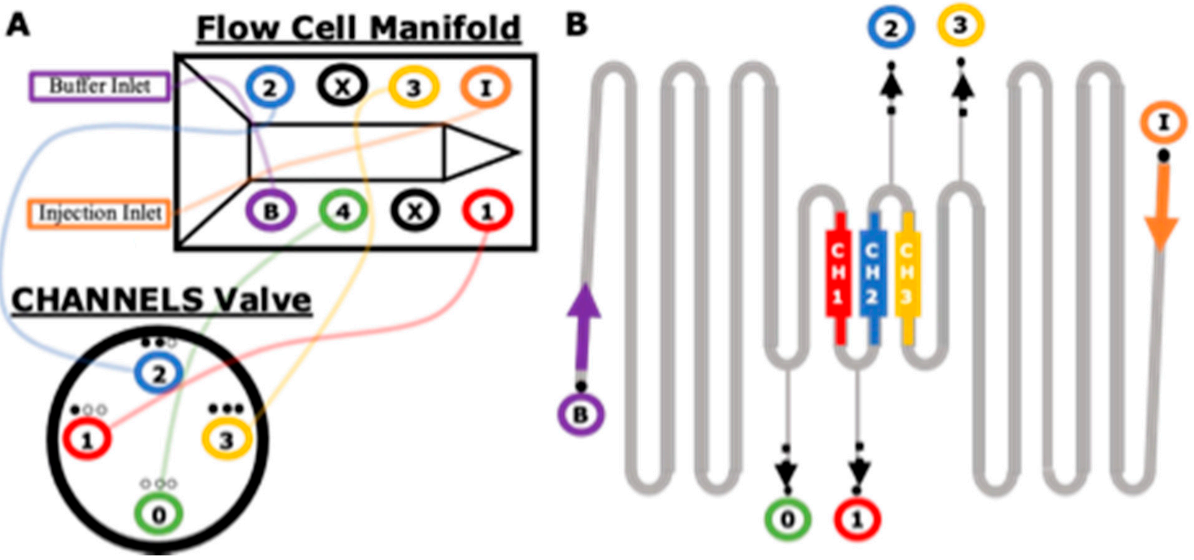

Figure S7. Flow system of the BI-2500 in reverse setup. (A) Plumbing schematic of the BI-2500 SPR system showing connections between the CHANNELS switch valve and the flow cell manifold in reverse setup. (B) Schematic of the flow cell showing the SPR channels CH1, CH2, and CH3. Injection and buffer flow in opposite directions from their respective inlets, meet at the junction of the selected outlet (based on CHANNELS valve position), and exit as waste.

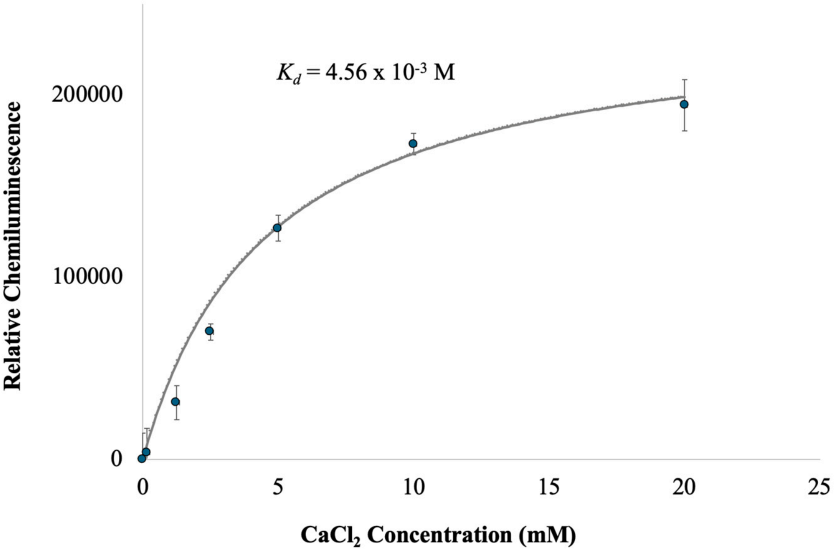

Figure S8. The effect of $CaCl_2$ on c-MYC-G3 chemiluminescence is hemin concentration-dependent. The chemiluminescence signal corrected for that determined at 0 mM $CaCl_2$ is plotted as a function of $CaCl_2$ concentration. Data was fit to a simple single-site binding equation (see Materials and Methods). Conditions: Tris buffer (25 mM), pH 8.0, hemin (0.05 μM), luminol (5 μM), Triton x-100 (0.0005%) and H_2O_2(1.3 mM). The calculated K_d is lower than that determined at higher (0.5 μM) hemin concentration (K_d = 7.19 mM).
